# Notes upon Diptheria

**Published:** 1860-02

**Authors:** J. H. Hollister

**Affiliations:** Professor of Physiology in Lind University


### NOTES UPON DIPTHERIA.

BY J. H. HOLLISTER, M. D.,
Professor of Physiology in Lind University.

At a recent meeting of the Chicago Medical Society, Dr. Hollister made a verbal report of some interesting cases of diptheria, accompanied by a specimen of false membrane of such remarkable development, that by vote of the Society, he was requested to furnish a written history of the case, with such notes upon the disease in question as he might deem proper. To which request he replies:

Gentlemen, I herewith present the history of the case of diptheria, to which I alluded when exhibiting a specimen of false membrane, at the last meeting of the Society.

The patient, George F., was a well formed, robust boy, aged 7 years. I first saw him at evening, October 4th, 1859. He had suffered severely from chills during the afternoon; febrile reaction was now apparent; the face was flushed; the skin hot and dry; tongue red, and but little furred; pulse 110; complained of head-ache and general languor. There was at this time no indication of local disease, and I suspected it merely the result of a sudden cold, perhaps attended with the congestion present in ague, and so frequently observed as complicating most diseases in that part of the city. I saw him again early, October 6th. He now complained of soreness of the throat; had the peculiar hoarse cough as in membranous croup; the neck, externally, was much swollen; the tonsils were much enlarged, and together with the adjacent structures, white with plastic lymph. The palate and anterior portions of the mouth were extremely red; the breathing hurried; pulse 120. Though at my first visit there was nothing of a local character complained of, there was now no chance of mistaking a well marked case of diptheria.

My prescription: ℞ Ipecac, grs. v.
Sub. Mur. Hyd. " x.
M. Div in Chart No. x.

Sig.—One every two hours.

Had reference to the sedative effect of the first and the power of the second, to prevent fibrilation of the effused lymph, and thereby favor its expulsion.

The throat was sponged every four hours with a solution.

℞ Argent Nitr ℈i.

Aqua Dist. ℥i. M.

Chlorate of Potash in solution was used freely as a gargle, and the external surface, a little distant from the trachea, subjected to counter irritation.

Twenty-four hours later, Oct. 7th, Prof. Davis saw him with me in consultation. The breathing was now more labored. I had previously produced emesis by the use of ipecac, with no apparent relief. The febrile symptoms had very much subsided. The blood was not duly oxidized, and he was evidently failing, with all the appearances of asphyxia. During the last twelve hours, I had detached firm patches of false membrane when using the sponge, some of them an inch in length.

It was suggested to use R. Sub. Sulph. of Mercury, grs. iv. one powder every four hours, with the two-fold view of dislodging the false membrane, and by its alterative effect, to pre vent fibrilation of the effused lymph.

At evening, after a terrific effort which nearly proved fatal, he succeeded in expelling a perfect tubular cast of the *trachea*, measuring seven inches in length, to the bifurcation, the exact representation of these and a tuft at the extremity of either one, which proved upon examination to be the bronchial subdivisions, thirteen in number on one side, and eleven on the other, each from one-fourth to one-half inch in length. *He now rested more quietly*, breathing with comparative ease, till near the morning of the 9th. We endeavored to sustain him as he seemed much exhausted. The imperfect aeration of blood was now very apparent. Twelve hours after the expulsion of the first specimen shown you, he threw off the second one, which was a likeness of the first, much less perfectly developed, and evidently from the upper portion of the trachea.

During the day his suffering was paroxysmal, at times rendering him almost frantic, permitting brief intervals of rest. His mental faculties seemed entirely unimpaired. He was fully conscious of all that occurred till three o'clock, P. M., when a suffocating paroxysm in a few moments ended the scene and the little sufferer was at rest.

A case of equal interest in the same family, but with very different symptoms, will be submitted at the next meeting.

